# Acute hemorrhagic necrotizing enterocolitis caused by non-O1/non-O139 *Vibrio cholerae* infection: A case report

**DOI:** 10.1097/MD.0000000000026460

**Published:** 2021-06-25

**Authors:** Mingjie Wu, Liangjing Zhou, Liping Cao

**Affiliations:** Department of General Surgery, Sir Run Run Shaw Hospital, School of Medicine, Zhejiang University, Hangzhou, Zhejiang province, China.

**Keywords:** acute hemorrhagic necrotizing enterocolitis, bacteremia, cirrhosis, colon necrosis, non-O1/non-O139 *Vibrio cholerae*

## Abstract

**Rationale:**

Acute hemorrhagic necrotizing enterocolitis (AHNE) is a rapidly progressive and extremely dangerous disease. Here we report a rare case caused by *Vibrio cholerae* (*V cholerae*).

**Patient concerns:**

A 70-year-old man was admitted to our emergency department because of a sudden loss of consciousness.

**Diagnoses:**

On admission with severe toxic shock, the patient presented with elevated body temperature, decreased blood pressure, abdominal tenderness and rebound pain, predominantly on the right side. Computed tomography showed swelling and thickening of the right colon and peritoneal effusion. Necrosis was found in the hepatic flexure of the colon. On the basis of these results, the patient was diagnosed with AHNE.

**Interventions and outcomes:**

After fluid resuscitation, an exploratory laparotomy was performed immediately. The procedure was successful. Despite antibiotic therapy, the patient's clinical condition progressively deteriorated and he died of multi-organ failure on day 3 after admission.

**Lessons:**

AHNE is a rapidly progressive and extremely dangerous disease. Here we report a case of AHNE caused by non-O1/non-O139 *V cholerae* infection. The clinical features, phenotypic analyses and the presence of a panel of known virulence genes in the isolated strain are described. To the best of our knowledge, this is the first report of *V cholerae* causing severe AHNE, which is of profound pedagogical significance.

## 1 Introduction

Acute hemorrhagic necrotizing enterocolitis (AHNE) is a rapidly progressive and extremely dangerous disease. Because it is clinically rare and lacks specific symptoms at the early stage, the misdiagnosis rate is 40% to 100% and the case fatality rate is up to 30%.^[1,2]^ The clinical manifestations are mainly abdominal pain and distension, diarrhea, hematochezia, vomiting, and fever. Bowel perforation, septic shock and other serious complications can occur in severe cases. So far, the etiology has not been established. It is considered that *Clostridium perfringens* type C is the main causative agent of AHNE, while streptococci, staphylococci, *Escherichia coli* and other bacteria reportedly invade the mucosa or submucosa thereby causing the disease.^[3,4]^ The present study is the first to report a case of *Vibrio cholerae* (*V cholera)* infection causing severe AHNE.

## 2 Case report

A 70-year-old man was admitted to our emergency department because of a sudden loss of consciousness. He had been presenting with asthenia for 1 day. Neither vomiting nor diarrhea was presented. The patient had a history of alcoholic cirrhosis.

On arrival, his body temperature was 38.1°C, blood pressure was 55/28mm Hg, and his heart rate was 124 beats per minute. The characteristic finding of the physical examination was tenderness and rebound pain over the whole abdomen, especially the right abdomen, suggesting acute peritonitis with circulatory shock. Laboratory tests revealed a significantly increased serum procalcitonin (116.8ng/ml), indicating a serious infection, whereas the white blood cell count (8.6 × 10^9^/L) and C-reactive protein level (10.6 mg/L) were not excessively high. Serum creatinine and serum potassium were 158 μmol/L and 2.5mmol/L, respectively. The pH value of blood was 7.229 and the bicarbonate level was 13.3mmol/L, suggesting metabolic acidosis.

**Figure 1:**
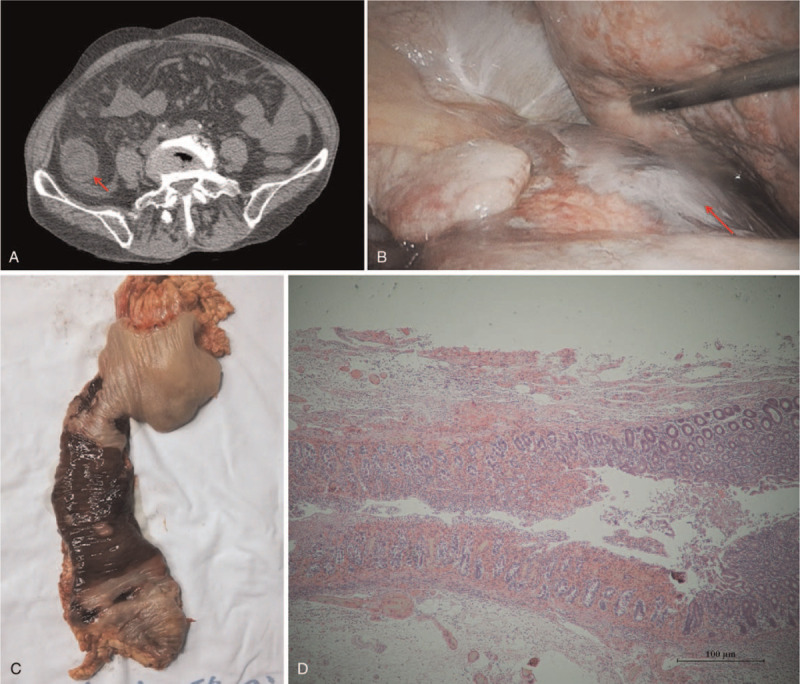
A: Abdominal CT showing swelling and thickening of the right colon and peritoneal effusion (arrow). B, C: Observation of necrosis in the hepatic flexure of the colon (arrow). D: Pathologic examination revealing large necrotic areas of colonic epithelium and abundant neutrophil infiltrations with abscess formation.

A computed tomography scan was performed, which showed swelling and thickening of the right colon and peritoneal effusion (Fig. 1A, arrow). After fluid resuscitation, broadspectrum antibiotics anti-infection, noradrenaline maintains blood pressure, and bowel rest, an exploratory laparotomy was performed immediately. Necrosis was found in the hepatic flexure of the colon (Fig. 1B and C, arrow). A pathologic examination revealed large necrotic areas of colonic epithelium and abundant neutrophil infiltrations with abscess formation (Fig. 1D). On the basis of these results, the patient was diagnosed with AHNE. After the exploratory laparotomy, the patient was given norepinephrine (0.2 μg/kg.min) to maintain blood pressure at 85–100/55–70 mm Hg, methylprednisolone to anti-inflammatory (30 mg/kg four times a day), broadspectrum antibiotics to strengthen anti-infection (Piperacillin Sodium Tazobactam Sodium 4.5 g three times a day and Vancomycin 1.0 g twice a day), and the respiratory support therapy.

After an incubation period, both the blood and necrotic tissue cultures yielded curved Gram-negative rods. The organisms were diagnosed as *V cholerae*. The organisms showed no agglutination with *V cholerae* O1 or O139 antisera. An *in vitro* susceptibility test showed this strain to be sensitive to all antibiotics tested. Despite antibiotic therapy, the patient's clinical condition progressively deteriorated and he died of multi-organ failure on day 3 after admission. For accurate identification of the strain isolated from the patient, we performed whole genome sequencing of this strain. The strain was found to be non-toxigenic, as it lacked the *ctxA*, *ctxB*, *tcpA,* and *toxR* genes. However, the strain carried other genes, such as *ompW*, *hlyA*, *hap,* and *rpoB*, which may play crucial roles in the pathogenicity of this strain.

## 3 Discussion

Cholera is an acute diarrheal disease caused by *V cholerae*, which can result in death due to dehydration within a few hours. *V cholerae* is now divided into more than 200 serogroups according to the different O antigens; only O1 and O139 *V cholerae* can generate epidemics of cholera.^[5]^ In recent years, reports of infections by non-O1/non-O139 *V cholerae* have been increasing.^[6]^ Infections caused by these bacteria are often associated with ingestion of contaminated seafood or exposure to coastal waters. The most common symptoms of such infections are mild to moderate gastroenteritis.^[7]^ However non-O1/non-O139 *V cholerae* are increasingly recognized as invasive pathogens in bacteremia and diseases other than acute diarrhea,^[8]^ particularly among immunosuppressed patients.

According to an epidemiological survey in Taiwan, about half of the patients infected with non-O1/non-O139 *V cholerae* manifested bacteremia.^[9]^ Without exception, these patients all had a history of hepatitis B cirrhosis, like the patient in the present report. The relevant mechanisms of the frequent occurrence of invasive *V cholerae* infections in patients with cirrhosis remain obscure; there are many hypotheses, such as decreased serum bactericidal activity, impaired filtration function in the cirrhotic liver, or increased serum iron levels,^[10]^ but the precise role of the defect in each mechanism requires further studies. Unlike other cases where patients only manifest acute gastroenteritis, these cirrhotic patients may present with severe toxic shock, with symptoms such as hypotension and increased serum procalcitonin. The prognosis for cirrhotic patients with *V cholerae* bacteremia was poor based on the epidemiological survey, in which 47% patients died.^[9]^ On the basis of bacteremia, patients may show various invasive infections, such as liver abscess,^[11]^ necrotizing fasciitis,^[12]^ meningitis,^[13]^ peritonitis,^[14]^ cellulitis and others. Zhou et al reported that the incidence of acute peritonitis was only 6.0%.^[15]^ In previous cases of cholera with acute peritonitis, all patients showed normal abdominal CT images and were usually diagnosed with spontaneous peritonitis.^[16,17]^ However in this case, the *V cholerae* infection caused severe colon necrosis and septic shock, eventually leading to the death of the patient. This was a rare case of adult necrotic colitis caused by *V cholerae* infection, which is worthy of clinical attention.

The mechanism that allows the strains to invade the bloodstream leading to necrosis has not been well elucidated. From the increasing full genome sequences available in the data libraries, we begin to understand that some non-O1 and non-O139 *V cholerae* strains encode for virulence factors that could influence their pathogenic potential.^[18,19]^ In this case, the strain carried genes such as *ompW*, *hlyA*, *hap*, *rpoB*, which may play crucial roles in the pathogenicity of this strain.

Necrotic enteritis is a group of acute intestinal segmental necrosis conditions with unknown etiology. One hypothesis is that bacteria colonization damages the intestinal mucosal barrier and causes arterial spastic ischemia, which is the main pathological process of necrotic enteritis. With disease progression, it can lead to whole-layer intestinal wall necrosis, perforation, peritonitis, sepsis and death. The affected patient may thus present with acute abdominal pain, abdominal distension, diarrhea, vomiting and blood in the stool. In severe cases, this can result in shock and high risk of death.

In conclusion, we learned in this case that non-O1/non-O139 *V cholerae* can also cause severe colon necrosis. Particularly we should consider how to identify patients with potentially severe AHNE, and further investigation is needed to elucidate the exact roles of the host, the environment and the pathogens in the pathogenesis of such invasive disease caused by non-O1/non-O139 *V cholerae* in both healthy and immunocompromised patients.

## Acknowledgments

The authors thank Amy Tong from Liwen Bianji, Edanz Editing China (www.liwenbianji.cn/ac), for editing the English text of a draft of this manuscript.

## Author contributions

**Conceptualization:** Mingjie Wu.

**Data curation:** Mingjie Wu, Liangjing Zhou.

**Investigation:** Mingjie Wu.

**Supervision:** Liping Cao.

**Writing – original draft:** Mingjie Wu.

**Writing – review & editing:** Mingjie Wu.
